# The double burden of malnutrition among adults in India: evidence from the National Family Health Survey-4 (2015-16)

**DOI:** 10.4178/epih.e2019050

**Published:** 2019-12-18

**Authors:** Mili Dutta, Y Selvamani, Pushpendra Singh, Lokender Prashad

**Affiliations:** 1 International Institute for Population Sciences (IIPS), Mumbai, India; 2 Department of Development Studies, International Institute for Population Sciences (IIPS), Mumbai, India; 3 Department of Humanities and Social Sciences, Indian Institute of Technology Roorkee, Roorkee, India; 4 Tata Institute of Social Sciences, Mumbai, India

**Keywords:** Thinness, Overweight, Obesity, Nutritional status, Socioeconomic factors, India

## Abstract

**OBJECTIVES:**

India still faces the burden of undernutrition and communicable diseases, and the prevalence of overweight/obesity is steadily increasing. The discourse regarding the dual burden of underweight and overweight/obesity has not yet been widely explored in both men and women. The present study assessed the determinants of underweight and overweight/obesity in India among adult men and women aged 15-49.

**METHODS:**

Population-based cross-sectional and nationally representative data from the National Family Health Survey-4 (2015-16), consisting of a sample of men and women, were analyzed. Stratified 2-stage sampling was used in the NFHS-4 study protocol. In the present study, bivariate and adjusted multinomial logistic regression analyses were performed to determine the correlates of underweight and overweight/obesity.

**RESULTS:**

The results suggested a persistently high prevalence of underweight coexisting with an increased prevalence of overweight/obesity in India. The risk of underweight was highest in the central and western regions and was also relatively high among those who used either smoking or smokeless tobacco. Overweight/obesity was more prevalent in urban areas, in the southern region, and among adults aged 35-49. Furthermore, level of education and wealth index were positively associated with overweight/obesity. More educated and wealthier adults were less likely to be underweight.

**CONCLUSIONS:**

In India, underweight has been prevalent, and the prevalence of overweight/obesity is increasing rapidly, particularly among men. The dual burden of underweight and overweight/obesity is alarming and needs to be considered; public health measures to address this situation must also be adopted through policy initiatives.

## INTRODUCTION

The rising prevalence of overweight/obesity—coexisting with undernutrition—is a significant health challenge in low-income and middle-income countries. On one hand, improved economic conditions, urbanization, the prevalence of sedentary lifestyles, and dietary changes have caused a steady increase in overweight/obesity [1-3]. On the other hand, many South Asian and sub-Saharan African countries are also facing challenges posed by undernutrition and its consequences [4]. In India, the share of underweight adults is the highest globally, even as the country experiences an ongoing rise in overweight/obesity [4,5]. Similarly, other South Asian countries such as Bangladesh and Nepal have a high proportion of underweight adults, along with a growing prevalence of overweight/obesity [6,7]. The presence of underweight coexisting with a rising prevalence of overweight/obesity is defined as the dual burden of malnutrition, and it poses major health challenges among the adult populations of developing countries, including India.

Both undernutrition and overnutrition are associated with adverse health outcomes. It is well-documented that overweight/obesity are significant predictors of overall mortality [8], chronic diseases such as diabetes [9], cardiovascular disease [10], multimorbidity [11,12], and disabilities [13]. Similarly, underweight is strongly associated with premature mortality, disabilities, and poor self-rated health and well-being, and this association is particularly strong in developing countries [14,15].

The determinants of the dual burden of malnutrition differ considerably based on gender, socioeconomic, and behavioural factors. Economic status has emerged as an important determinant of overweight/obesity, particularly in developing countries [15-18]. In contrast, less educated and socially disadvantaged adults tend to be underweight [19,20]. Studies also suggest that several risk factors are associated with low and high body mass index (BMI), including physical inactivity, poor diet, and tobacco use [21-23].

In India, few studies have examined the relationships among socioeconomic status (SES), tobacco use, dietary patterns, and BMI. Furthermore, most studies of BMI have focused on women respondents. However, it is also necessary to examine changing patterns in men’s body weight in order to understand the impact of modifiable health factors on men. Furthermore, considerable variations in BMI have been noted across states in India [24,25], as these states exhibit diverse and unequal patterns of economic development, and demographic and epidemiological transitions are occurring differently across different regions [26,27]. Therefore, it is necessary to understand the existence of the dual burden of undernutrition and overnutrition among the population (men and women) of India. In this context, the present study assesses the determinants of underweight and overweight/obesity in India for both men and women.

## MATERIALS AND METHODS

### Data source

**Figure 1.**
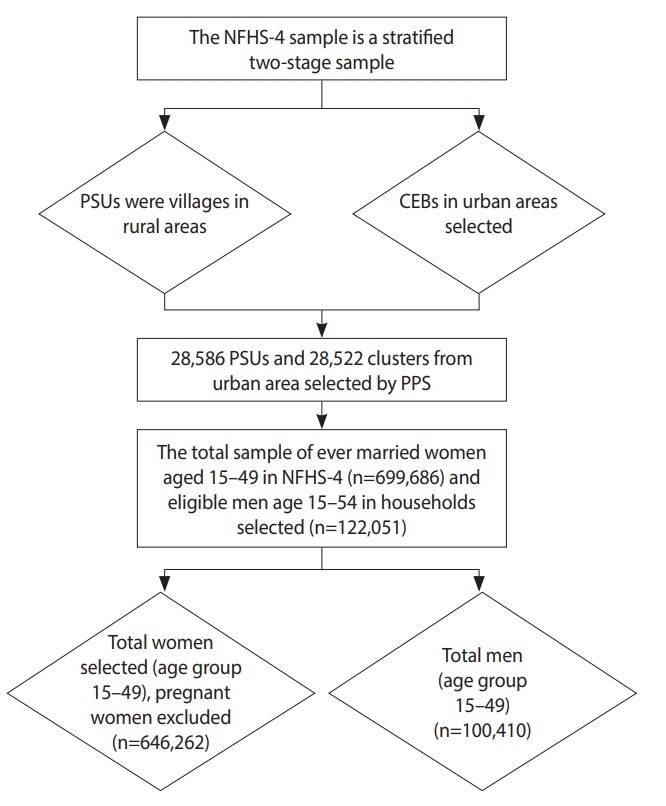
Flow chart for the sampling and sample selection, NFHS-4, 2015/16, India. NFHS, National Family Health Survey; PSUs, primary sampling units; CEB, census enumeration block; PPS, probability proportional to size.

The present study used data from the National Family Health Survey (NFHS-4), the fourth in the series of NFHS. The survey was conducted in 2015-16 and obtained information about population, nutrition, and health for the states of India and for the country as a whole. The NFHS-4 was conducted by interviewing randomly-selected women aged 15-49 and men aged 15-54. Stratified 2-stage sampling was used as the sampling design for the NFHS-4 study. In the first stage, the primary sampling units were selected, and in the second stage, the households for the study were selected. Primary sampling units with at least 300 households were divided into segments of approximately 100-150 households. Two of the segments were selected using systematic sampling with probability proportional to size. From each selected rural and urban cluster, 22 households were selected using systematic sampling (Figure 1).

### Measures

#### Dependent variable

BMI is defined as the individual’s weight in kilograms divided by the square of the individual’s height in meters (kg/m^2^). In this study, objectively measured height and weight were used to calculate BMI. We followed World Health Organization standards for the categorisation of BMI. Subjects were categorised into the following 3 groups according to BMI: underweight, less than 18.5 kg/m^2^; normal, between 18.5 and 24.9 kg/m^2^; and overweight/obesity, equal to or greater than 25.0 kg/m^2^ [28].

#### Predictor variables

The study population was categorised into 4 groups by education level: no education, primary education, secondary education, and more than secondary education.

The wealth index was calculated using the number and type of consumer goods owned by a household, as well as housing characteristics. Each household asset was allocated a weight or factor score measure through principal component analysis, and values were standardized in relation to a normal distribution. The sample was then divided into quintiles: poorest, poorer, middle, richer, and richest.

Subjects were categorised into 4 groups according to their tobacco use: no tobacco, only smoking tobacco (cigarettes, bidis, cigars, or hookah pipes), only smokeless tobacco (*paan masala* or *gutkha, khaini, paan* with tobacco, any other type of chewing tobacco, or snuff), and both smoking and smokeless tobacco.

To identify the impact of food consumption on underweight and overweight/obesity, the frequency of intake of various food items was obtained. Respondents could rate their food intake of each item as never, occasionally, or daily.

The regions of India were divided into 6 categories: northern, central, eastern, western, northeastern, and southern. The northern region consisted of Chandigarh, Delhi, Haryana, Himachal Pradesh, Jammu and Kashmir, Punjab, Rajasthan, and Uttarakhand. The central region included Chhattisgarh, Madhya Pradesh, and Uttar Pradesh, and the eastern region included Jharkhand, Odisha, West Bengal, and Bihar. The northeastern region included Nagaland, Sikkim, Arunachal Pradesh, Manipur, Mizoram, Tripura, Meghalaya, and Assam; the western region included Dadra and Nagar Haveli, Daman and Diu, Gujarat, Maharashtra, and Goa. Lastly, the southern region consisted of Andaman and Nicobar Islands, Andhra Pradesh, Karnataka, Kerala, Tamil Nadu, Telangana, Puducherry, and Lakshadweep.

#### Covariates

The selected covariates included age group (15-19, 20-34, and 35-49), place of residence (rural or urban), caste (Scheduled Caste, Scheduled Tribe, Other Backward Class, or other), religion (Hindu, Muslim, or other), currently employed (no or yes), and marital status (unmarried, currently married, widowed, or divorced/separated).

### Statistical analysis

In this study, bivariate analysis was performed to determine the relationship of background characteristics with the weighted prevalence of underweight and overweight/obesity. State-level differences in underweight and overweight/obesity were also assessed. In addition, multinomial logistic regression analysis was used to evaluate the determinants of underweight and overweight/obesity. Relative risk (RR) ratios were presented with confidence intervals (CIs). Moreover, separate analyses were conducted for men and women. The analysis was restricted to women aged 15-49 who were not pregnant and who had not given birth within 2 months prior to the survey (n=646,262) and men aged 15-49 (n=100,410). All data analysis was carried out using Stata version 14.0 (StataCorp., College Station, TX, USA).

### Ethics statement

This research does not have an ethical code because the data source used in this study gathered from publically available data and these data considered as secondary data.

## RESULTS

**Figure 2.**
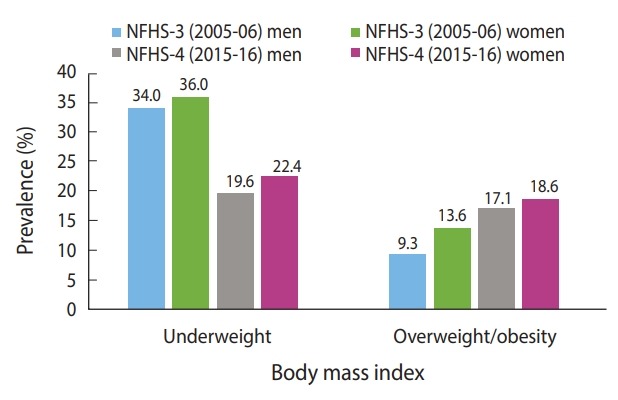
Distribution of underweight and overweight/obesity among men and women in India, National Family Health Survey (NFHS)- 2005/06-2015/16.

During the study period, the prevalence of underweight decreased and the prevalence of overweight/obesity increased among both men and women (Figure 2). The prevalence of both underweight and overweight/obesity were higher among women than men. The prevalence of overweight/obesity among men almost doubled from 2005-06 to 2015-16.

**Figure 3.**
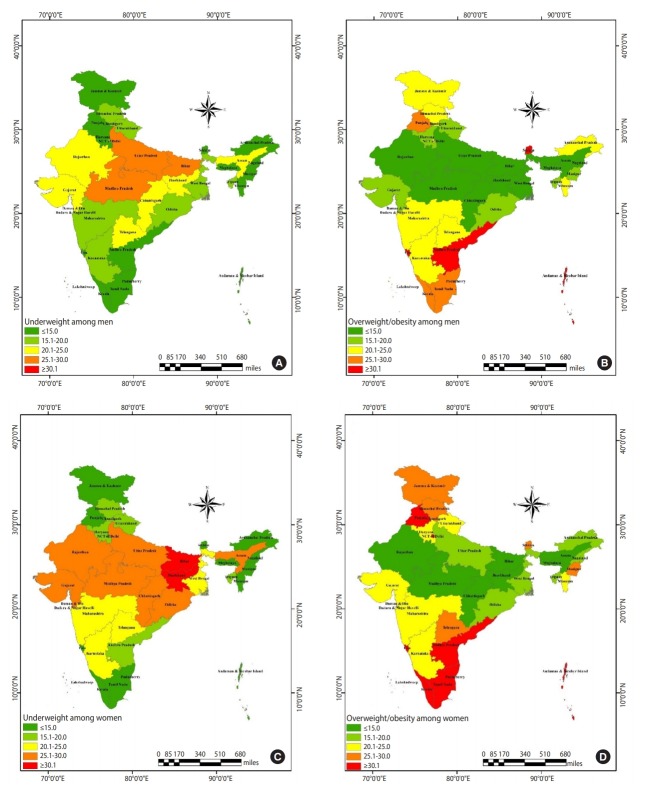
Distribution of underweight and overweight/obesity among men (A, B) and women (C, D) across the states of India, National Family Health Survey-4, 2015/16, India.

Figure 3A and 3B shows the distribution of underweight and overweight/obesity among men in different Indian states. Among men, a higher prevalence of underweight was clustered in Uttar Pradesh, Bihar, and Madhya Pradesh. In contrast, the prevalence was low in the southern and northern regions, as well as in some of the northeastern region. A high prevalence of overweight/obesity was found in Goa, Andhra Pradesh, and Sikkim, where more than 30% of men were overweight/obese; this was followed by Kerala, Tamil Nadu, and Punjab, where 25% to 30% of men were found to be overweight/obese. Telangana was the state with the most similar prevalence (20 to 25%) of underweight and overweight/obesity.

Figure 3C and 3D shows the prevalence of underweight and overweight/obesity among women in the Indian states. Bihar and Jharkhand displayed a higher prevalence of underweight than the other states. Furthermore, a relatively high risk of underweight was found to cluster in the middle part of India, including Rajasthan, Gujarat, Madhya Pradesh, Chhattisgarh, Odisha, Uttar Pradesh, and Assam. Figure 3D shows a lower prevalence of overweight/obesity in the abovementioned states. Overweight/obesity was found to be highly clustered in the southern states of India. In addition, Punjab, Goa, and Delhi displayed a high prevalence of overweight/obesity. Maharashtra and Karnataka showed a similar prevalence for both underweight and overweight/obesity.

Table 1 illustrates the prevalence, by percentage, of underweight and overweight/obesity depending on background characteristics. The prevalence of underweight was highest among men and women in the 15-year to 19-year age group, while overweight/obesity was highest among men and women aged 35-49 years. The prevalence of overweight/obesity was highest in the southern region for both men and women. Underweight was more prevalent in rural areas, whereas overweight/obesity was higher in urban areas. The prevalence of underweight was highest among Scheduled Tribe members, Hindus, those who had never married, those who were less educated, and those in the poorest quintile for both men and women. The prevalence of overweight/obesity was highest among men and women belonging to “other” castes and religions, those with more than a secondary education, those with a high wealth index, and non-tobacco consumers. Unemployed men were more likely than employed men to be underweight, but the employment status of women did not make a significant difference in the prevalence of underweight. Moreover, the prevalence of overweight/obesity was higher among employed than unemployed men, but the reverse was true for women. The prevalence of underweight and overweight/obesity according to food consumption is shown in Table 2. As shown in the Table 3, individuals were typically more likely to be overweight/obesity if the assessed foods were consumed daily, whereas underweight was more prevalent if the foods were never consumed. The estimated predictive power of food consumption for underweight and overweight/obesity risk is presented in Table 4.

### Estimates of multinomial logistic regression to identify factors affecting body mass index in India (National Family Health Survey 2015-16)

The factors affecting underweight and overweight/obesity in men and women according to background characteristics are shown in Table 3. The likelihood of being underweight decreased as age increased, while the likelihood of overweight/obesity tended to increase with age among both men and women. Men aged 35-49 years were 3.15 times more likely to be overweight/obese than man subjects aged 15-19 years. Similarly, women aged 35-49 were 4.52 times more likely to be overweight/obese than woman respondents aged 15-19 years. Compared to northern region, men in all regions except for the central region were more likely to be overweight/obese. In contrast, the likelihood of overweight/obesity was higher among women in the western, and southern regions than among women in the northern region. Men who lived in urban areas were 1.18 times more likely to be overweight/obese than men residing in rural regions. Women in urban areas were 10.5% less likely to be underweight and 1.28 times more likely to be overweight/obese than women living in rural areas. Regarding caste, Scheduled Tribe members were least likely to be overweight/obese, whereas people of “other” castes were most likely to be overweight/obese for both men and women.

Men who were currently employed were less likely than unemployed men to be underweight, and employed women were less likely than unemployed women to be overweight/obesity. Furthermore, with regard to marriage status, married men and women were least likely to be underweight, and the likelihood of being overweight/obesity was much higher in this group than in other groups. As education level increased, the prevalence of underweight tended to decrease, while the prevalence of overweight/obesity tended to increase. Compared to less wealthy individuals, individuals with a higher wealth index were more likely to be overweight/obesity and less likely to be underweight. Those in the highest wealth quintile for men and women were 5.10 and 5.63 times more likely than those in the lowest wealth quintile, respectively, to be overweight/obese. The use of tobacco increased the likelihood of underweight among both men and women, but it decreased the likelihood of overweight/obesity.

The likelihood of underweight decreased with occasional and daily consumption of milk among men, while among women, it decreased with daily milk consumption (Table 4). Further, the occasional and daily consumption of pulses decreased the likelihood of overweight/obesity among women. In addition, the occasional and daily intake of vegetables and fruit decreased the risk of underweight among women. However, the daily intake of fruit was associated with increased risk of overweight/obesity among men. The occasional consumption of fish increases the risk of overweight/obesity. Moreover, the daily and occasional intake of aerated drinks significantly increased the risk of overweight/obesity among men and women.

## DISCUSSION

Using recent data from the NFHS-4, this study examined the determinants of undernutrition and overnutrition among adult men and women aged 15-49 in India. In particular, the aim was to understand the association of SES, diet, and tobacco use on underweight and overweight/obesity. We additionally studied variations in BMI across the states of India.

We noticed a sharp rise in the prevalence of overweight/obesity between 2005-2006 and 2015-2016. Nevertheless, the proportion of men (19.6%) and women (22.4%) studied who were underweight, thus suggesting the presence of the dual burden of malnutrition among the adult population in India. It was also found that underweight was prevalent in most of the Empowered Action Group states, which are states that lag behind in various demographic and social indicators. However, the southern states displayed a high prevalence of overweight/obesity. Further, the prevalence of underweight and overweight/obesity was found to be dependent on SES. In particular, the association between wealth quintile and overweight/obesity was highly significant and positive for men and women. The highest percentage of underweight subjects was observed among adults with the poorest SES.

The results of this study are consistent with previous findings that showed a higher prevalence of underweight among adults with low SES [20]. Similarly, overweight is more prevalent among educated and wealthier people [16-18]. Previous studies conducted in India have identified SES as an important factor involved in access to food, which can influence body weight. Higher SES also appears to be strongly correlated with physical inactivity, which subsequently plays an important role in determining body weight [16]. Additionally, gender differences in BMI are notable. Though the prevalence of obesity was higher among women respondents, the gender gap in overweight/obesity is gradually declining. The gender differences in overweight/obesity in India can be attributed to health risk factors, such as lower physical activity (PA) among women respondents [21].

In this study, tobacco use was found to be significantly associated with BMI. This association was positive with underweight and negative with overweight/obesity, and the results are in line with those of previous studies conducted in India [22,23]. The strength of the association between tobacco use and underweight has been found to be stronger for women respondents than men. The existing literature has identified tobacco use as a main cause of appetite suppression, which is reflected in reduced food intake and lower BMI [29-31]. Tobacco is smoked more commonly by adults of low SES, which raises concern about tobacco use, as it has shown a strong association with various adverse health outcomes, including mortality.

Behavioural risk factors, such as the intake of milk/curd, vegetables, fruit, fish, eggs, and aerated drinks, were found to be associated with BMI. The previous literature has shown that frequent consumption of vegetables and dairy products is positively associated with BMI [1]. The consumption of pulses, milk, and vegetables has been shown to reduce the risk of underweight, and high consumption of fish and aerated drinks increases the risk of overweight/obesity [32].

The coexistence of underweight and overweight/obesity in developing countries like India calls for attention. Proactive measures to prevent underweight and overweight/obesity are important. The results from this study strongly suggest that overweight/obesity is prevalent among adults of higher SES, thus suggesting possible measures to promote changes in dietary patterns and PA. In this study, tobacco use emerged as a significant factor in determining BMI, and the prevalence of tobacco use is high in India. In this context, creating awareness about the implications of tobacco use for nutritional status is important. Furthermore, measures to promote PA are also important. In India, more than half of adults aged 20 or above are physically inactive [21]. In particular, women are at a higher risk than men of being inactive. In this context, measures to promote PA will have significant implications for reducing the burden of overweight/obesity and chronic conditions. In future research, the reasons for low levels of PA in women, the reasons why high-income people tend to be overweight/obese, and public health strategies to address these problems should be investigated.

The limitations of this study include the cross-sectional nature of the data used. Therefore, no causal relationships can be established. Furthermore, we used only underweight and overweight/obesity as measures of nutritional status. Additionally, NFHS provides no information on several other risk factors for underweight and overweight/obesity, such as PA, sleep patterns, and amount of stress.

However, the main strength of this study is that the data used were large-scale and nationally representative. Therefore, the results of this study can be generalized to the national level. Furthermore, most of the previous literature has focused mainly on correlates of BMI for women of reproductive age. This study provided information on the prevalence of underweight and overweight/ obesity across the states of India for both men and women and their major correlates.

The findings suggest that the dual burden of malnourishment persists in India. Underweight is already prevalent in India, and the prevalence of overweight/obesity is increasing rapidly, particularly among men. The study further showed a high clustering of underweight in most of the Empowered Action Group states, whereas overweight/obesity was mostly clustered in the southern states. The results of this study suggest significant associations between socioeconomic variables and underweight. The major risk factor for underweight was found to be tobacco use; in particular, the use of smoking tobacco was associated with underweight status. However, adults aged 35-49, those residing in the northeastern region, those who were currently employed and more highly educated, and those who had a higher wealth index were at lower risks of being underweight. In contrast, older adults, those residing in the western and southern regions, those residing in urban areas, and more educated and wealthier individuals were more prone to being overweight/obese. We found little difference in the risk factors for underweight and overweight/obesity with respect to gender in India. The dual burden of underweight and overweight/obesity is alarming and must be taken into consideration so that public health measures can be implemented through policy initiatives. From this perspective, measures to prevent tobacco use and overweight/obesity-related interventions may enhance the nutritional status of the adult population in India.

## Figures and Tables

**Table 1.** Distribution of underweight and overweight/obesity by socioeconomic characteristics, NFHS-4, 2015-16, India

Characteristics^1^	Men, %			Women, %		
	Underweight	Overweight/obesity	Total (n)	Underweight	Overweight/ obesity	Total (n)
Age (yr)^***^						
15-19	44.9	4.8	18,493	42.0	4.3	117,924
20-34	16.6	17.7	45,927	22.8	17.9	294,396
35-49	12.3	27.6	35,990	14.0	32.2	233,942
Region^***^						
Northern	16.5	19.5	22,161	19.4	22.2	129,946
Central	26.4	11.9	25,446	26.4	15.4	170,828
Eastern	22.2	13.8	15,433	26.7	15.4	116,030
Western	21.3	22.3	10,901	24.7	23.7	52,276
Northeastern	18.2	14.0	12,922	22.0	14.7	91,146
Southern	14.2	27.5	13,547	17.0	29.7	86,036
Place of residence^***^						
Rural	23.0	14.3	69,022	26.8	15.1	456,728
Urban	15.5	26.6	31,388	15.5	31.4	189,534
Caste^***^						
SC	22.9	15.0	17,963	25.3	17.3	114,935
ST	25.1	9.8	17,887	31.8	10.1	116,898
OBC	20.3	19.6	39,059	23.0	20.9	253,061
Other	16.6	24.7	19,919	17.8	27.4	131,157
Religion^***^						
Hindu	20.7	18.4	74,920	23.6	19.9	480,743
Muslim	19.3	19.9	13,886	21.6	23.7	86,135
Other	13.9	24.5	11,604	16.4	26.5	79,384
Currently employed^***^						
No	32.8	11.1	25,772	22.7	22.2	85,597
Yes	16.1	21.5	74,638	22.7	20.6	27,239
Marital status^***^						
Never married	30.7	10.2	38,688	37.4	6.6	167,696
Married	13.6	24.5	60,458	18.4	25.2	450,995
Widowed	24.2	13.5	577	18.1	26.5	20,000
Divorced/separated	22.3	15.4	687	20.2	22.7	7,571
Education level^***^						
Not educated	22.6	12.0	12,251	24.7	16.9	183,128
Up to primary	21.7	16.4	12,335	22.0	21.8	81,509
Up to secondary	22.3	18.0	59,890	24.0	21.4	308,908
More than secondary	10.4	28.5	15,934	16.0	25.9	72,717
Wealth index^***^						
Poorest	31.9	4.8	16,639	36.0	5.9	121,389
Poorer	26.6	9.8	21,039	29.6	11.4	137,673
Middle	20.5	16.6	21,970	23.1	18.8	136,665
Richer	16.2	24.4	20,798	17.1	28.2	129,062
Richest	10.6	32.7	19,964	11.6	36.2	121,473
Tobacco use^***^						
No tobacco	20.9	21.0	51,663	22.5	21.2	577,413
Only smoking tobacco	16.6	19.2	15,473	31.9	12.2	5,462
Only smokeless tobacco	20.8	15.7	22,335	28.4	15.0	60,988
Both smoking and smokeless tobacco	20.1	13.2	10,939	26.1	13.0	2,399

NFHS, National Family Health Survey; SC, Scheduled Caste; ST, Scheduled Tribe; OBC, Other Backward Class.

1 Significance level is obtained from chi-square test.

*** p<0.001.

**Table 2.** Distribution of underweight and overweight/obesity by food consumption, NFHS-4, 2015-16, India

Food consumption^1^	Men, %			Women, %		
	Underweight	Overweight/ obesity	Total (n)	Underweight	Overweight/ obesity	Total (n)
Milk/curd^***^						
Never	26.3	12.8	6,045	27.7	17.8	55,707
Occasionally	22.6	16.0	51,468	25.9	17.2	332,412
Daily	17.0	22.7	42,897	19.0	25.0	258,143
Pulses^***^						
Never	21.7	14.6	456	28.9	19.9	3,586
Occasionally	21.2	17.9	55,127	23.7	19.9	367,030
Daily	19.0	20.1	44,827	21.9	21.8	275,646
Vegetables^***^						
Never	25.6	12.9	418	30.6	16.8	2,117
Occasionally	21.5	18.0	52,730	23.9	20.0	326,903
Daily	18.6	20.1	47,262	21.8	21.7	317,242
Fruits^***^						
Never	26.9	9.9	2015	30.1	14.0	15,451
Occasionally	20.8	18.0	88,024	23.9	19.4	563,351
Daily	14.2	28.4	10,371	14.5	31.8	67,460
Eggs^***^						
Never	21.4	18.7	20,378	23.2	20.8	19,825
Occasionally	20.1	18.6	75,671	23.1	20.3	429,937
Daily	16.5	24.3	4,361	17.7	27.7	20,500
Fish^***^						
Never	21.2	18.3	28,481	23.3	20.5	233,755
Occasionally	20.2	18.9	67,796	23.5	20.3	385,262
Daily	13.6	23.4	433	14.6	27.6	27,245
Chicken^***^						
Never	21.2	18.3	23 521	23.1	20.6	211,694
Occasionally	19.9	19.1	75 153	23.0	20.7	426,757
Daily	18.8	21.0	1736	17.7	28.0	7,811
Fried food^***^						
Never	19.5	20.2	7,659	22.1	24.1	29,181
Occasionally	20.3	18.9	81,140	23.1	20.5	540,818
Daily	19.8	18.1	11,611	21.7	21.0	76,263
Aerated drinks^***^						
Never	21.0	16.2	12,549	25.9	17.8	106,120
Occasionally	20.3	19.0	81,401	22.5	21.3	509,147
Daily	17.2	22.8	6,460	20.3	22.3	30,995

NFHS, National Family Health Survey.

1 Significance level is obtained from chi-square test.

*** p<0.001.

**Table 3.** Adjusted relative risk ratio of underweight and overweight/obesity by socioeconomic characteristics as determined through multinomial logistic regression analysis, NFHS-4, 2015-16, India^1^

Characteristics	Men		Women	
	Underweight	Overweight/obesity	Underweight	Overweight/obesity
Age (yr)				
15-19	1.00 (reference)	1.00 (reference)	1.00 (reference)	1.00 (reference)
20-34	0.40 (0.38, 0.42)^***^	2.14 (1.95, 2.34)^***^	0.68 (0.65, 0.72)^***^	2.30 (2.10, 2.53)^***^
35-49	0.34 (0.32, 0.37)^***^	3.15 (2.85, 3.48)^***^	0.44 (0.41, 0.47)^***^	4.52 (4.11, 4.98)^***^
Region				
Northern	1.00 (reference)	1.00 (reference)	1.00 (reference)	1.00 (reference)
Central	1.32 (1.25, 1.40)^***^	0.89 (0.83, 0.94)^***^	1.08 (1.02, 1.13)^***^	0.99 (0.93, 1.04)
Eastern	1.04 (0.98, 1.11)	1.17 (1.09, 1.26)^***^	1.15 (1.09, 1.22)^***^	1.06 (0.98, 1.13)
Western	1.58 (1.48, 1.70)^***^	1.22 (1.14, 1.31)^***^	1.62 (1.52, 1.72)^***^	1.14 (1.07, 1.22)^***^
Northeastern	0.65 (0.60, 0.71)^***^	1.29 (1.19, 1.40)^***^	0.60 (0.55, 0.65)^***^	1.06 (0.98, 1.15)
Southern	0.98 (0.91, 1.05)	1.57 (1.47, 1.68)^***^	1.07 (1.00, 1.14)^**^	1.44 (1.36, 1.54)^***^
Place of residence				
Rural	1.00 (reference)	1.00 (reference)	1.00 (reference)	1.00 (reference)
Urban	1.01 (0.96, 1.05)	1.18 (1.13, 1.23)^***^	0.89 (0.85, 0.93)^***^	1.28 (1.23, 1.33)^***^
Caste				
SC	1.00 (reference)	1.00 (reference)	1.00 (reference)	1.00 (reference)
ST	0.79 (0.75, 0.84)^***^	0.83 (0.77, 0.90)^***^	0.974 (0.92, 1.03)	0.76 (0.71, 0.81)^***^
OBC	0.97 (0.92, 1.01)	1.07 (1.01, 1.13)^**^	0.967 (0.93, 1.01)	1.02 (0.97, 1.07)
Other	0.87 (0.82, 0.92)^***^	1.22 (1.15, 1.30)^***^	0.88 (0.83, 0.93)^***^	1.13 (1.07, 1.20)^***^
Religion				
Hindu	1.00 (reference)	1.00 (reference)	1.00 (reference)	1.00 (reference)
Muslim	0.93 (0.88, 0.99)^**^	0.99 (0.93, 1.06)	0.91 (0.86, 0.96)^***^	1.29 (1.22, 1.37)^***^
Other	0.69 (0.63, 0.74)^***^	1.15 (1.07, 1.23)^***^	0.60 (0.56, 0.64)^***^	1.09 (1.02,1.16)^***^
Currently employed				
No	1.00 (reference)	1.00 (reference)	1.00 (reference)	1.00 (reference)
Yes	0.72 (0.69, 0.75)^***^	1.00 (0.94, 1.06)	0.96 (0.93, 1.00)^**^	0.93 (0.89, 0.97)^***^
Marital status				
Never married	1.00 (reference)	1.00 (reference)	1.00 (reference)	1.00 (reference)
Married	0.76 (0.72, 0.80)^***^	1.91 (1.80, 2.02)^***^	0.64 (0.61, 0.67)^***^	2.19 (2.05, 2.34)^***^
Widowed	1.14 (0.92, 1.41)	1.34 (1.02, 1.78)^**^	0.69 (0.62, 0.77)^***^	2.00 (1.79, 2.23)^***^
Divorced/separated	1.06 (0.86, 1.31)	1.31 (1.03, 1.66)^**^	0.83 (0.72, 0.96)^**^	1.90 (1.62, 2.23)^***^
Education level				
Not educated	1.00 (reference)	1.00 (reference)	1.00 (reference)	1.00 (reference)
Up to primary	1.03 (0.97, 1.11)	1.20 (1.10, 1.30)^***^	0.89 (0.84, 0.94)^***^	1.15 (1.09, 1.22)^***^
Up to secondary	0.87 (0.83, 0.93)^***^	1.23 (1.15, 1.32)^***^	0.86 (0.82, 0.90)^***^	1.19 (1.13, 1.25)^***^
More than secondary	0.59 (0.54, 0.64)^***^	1.38 (1.27, 1.50)^***^	0.71 (0.66, 0.76)^***^	1.04 (0.97, 1.12)
Wealth index				
Poorest	1.00 (reference)	1.00 (reference)	1.00 (reference)	1.00 (reference)
Poorer	0.85 (0.80, 0.89)^***^	1.61 (1.47, 1.76)^***^	0.84 (0.81, 0.89)^***^	1.75 (1.62, 1.90)^***^
Middle	0.68 (0.65, 0.72)^***^	2.49 (2.27, 2.72)^***^	0.73 (0.69, 0.76)^***^	2.76 (2.55, 2.99)^***^
Richer	0.59 (0.56, 0.64)^***^	3.69 (3.37, 4.04)^***^	0.60 (0.57, 0.64)^***^	4.33 (3.99, 4.71)^***^
Richest	0.43 (0.40, 0.47)^***^	5.10 (4.63, 5.62)^***^	0.47 (0.44, 0.51)^***^	5.63 (5.15, 6.15)^***^
Tobacco				
No tobacco	1.00 (reference)	1.00 (reference)	1.00 (reference)	1.00 (reference)
Only smoking tobacco	1.09 (1.03, 1.15)^***^	0.78 (0.74, 0.83)^***^	1.49 (1.25, 1.77)^***^	1.02 (0.83, 1.25)
Only smokeless tobacco	1.05 (1.00, 1.10)^**^	0.85 (0.81, 0.89)^***^	1.37 (1.30, 1.45)^***^	0.88 (0.83, 0.95)^***^
Both smoking and smokeless tobacco	1.04 (0.98, 1.11)	0.73 (0.68, 0.78)^***^	1.06 (0.77, 1.45)	0.80 (0.61, 1.05)

Values are presented as relative risk (95% confidence interval). NFHS, National Family Health Survey; SC, Scheduled Caste; ST, Scheduled Tribe; OBC, Other Backward Class.

1 The results were adjusted for age, marital status, caste, religion, place of residence, schooling, wealth quintile, employment status, and tobacco use.

** p<0.01,

*** p<0.001.

**Table 4.** Adjusted relative risk ratio of underweight and overweight/obesity by food consumption patterns as determined through multinomial logistic regression analysis, NFHS-4, 2015-16, India^1^

Food consumption	Men		Women	
	Underweight	Overweight/obesity	Underweight	Overweight/obesity
Milk/curd				
Never	1.00 (reference)	1.00 (reference)	1.00 (reference)	1.00 (reference)
Occasionally	0.82 (0.76, 0.88)^***^	0.94 (0.86, 1.03)	1.02 (0.96, 1.08)	0.97 (0.91, 1.04)
Daily	0.75 (0.69, 0.81)^***^	0.99 (0.90, 1.08)	0.92 (0.86, 0.98)^***^	0.94 (0.88, 1.01)^*^
Pulses				
Never	1.00 (reference)	1.00 (reference)	1.00 (reference)	1.00 (reference)
Occasionally	1.03 (0.80, 1.34)	1.17 (0.85, 1.61)	1.04 (0.83, 1.29)	0.72 (0.57, 0.92)^***^
Daily	1.05 (0.81, 1.36)	1.24 (0.90, 1.71)	1.02 (0.82, 1.27)	0.73 (0.57, 0.94)^**^
Vegetables				
Never	1.00 (reference)	1.00 (reference)	1.00 (reference)	1.00 (reference)
Occasionally	0.90 (0.71, 1.14)	1.08 (0.78, 1.51)	0.76 (0.59, 0.98)^**^	0.94 (0.68, 1.30)
Daily	0.85 (0.67, 1.08)	1.14 (0.82, 1.59)	0.73 (0.57, 0.94)^**^	1.05 (0.76, 1.45)
Fruits				
Never	1.00 (reference)	1.00 (reference)	1.00 (reference)	1.00 (reference)
Occasionally	1.02 (0.91, 1.14)	1.10 (0.93, 1.29)	0.91 (0.82, 1.00)^*^	1.01 (0.88, 1.16)
Daily	1.01 (0.89, 1.15)	1.25 (1.05, 1.49)^**^	0.82 (0.73, 0.92)^***^	1.13 (0.98, 1.31)
Eggs				
Never	1.00 (reference)	1.00 (reference)	1.00 (reference)	1.00 (reference)
Occasionally	0.92 (0.86, 0.98)^**^	0.93 (0.86, 1.01)^*^	0.98 (0.92, 1.04)	0.89 (0.83, 0.96)^***^
Daily	1.02 (0.91, 1.15)	1.02 (0.91, 1.15)	1.16 (1.03, 1.30)^**^	1.00 (0.90, 1.12)
Fish				
Never	1.00 (reference)	1.00 (reference)	1.00 (reference)	1.00 (reference)
Occasionally	1.02 (0.96, 1.09)	1.10 (1.02, 1.19)^**^	0.98 (0.92, 1.04)	1.07 (0.99, 1.15)^*^
Daily	0.84 (0.74, 0.96)^***^	1.04 (0.92, 1.17)	0.78 (0.70, 0.87)^***^	1.08 (0.98, 1.20)
Chicken				
Never	1.00 (reference)	1.00 (reference)	1.00 (reference)	1.00 (reference)
Occasionally	0.95 (0.88, 1.03)	1.07 (0.98, 1.17)	0.99 (0.92, 1.07)	1.06 (0.98, 1.16)
Daily	1.09 (0.91, 1.30)	1.09 (0.92, 1.29)	0.99 (0.83, 1.20)	1.07 (0.90, 1.27)
Fried food				
Never	1.00 (reference)	1.00 (reference)	1.00 (reference)	1.00 (reference)
Occasionally	1.00 (0.93, 1.07)	0.96 (0.89, 1.03)	1.06 (0.98, 1.15)	0.86 (0.79, 0.94)^***^
Daily	1.01 (0.92, 1.10)	0.93 (0.85, 1.02)	1.03 (0.94, 1.14)	0.89 (0.81, 0.98)^**^
Aerated drinks				
Never	1.00 (reference)	1.00 (reference)	1.00 (reference)	1.00 (reference)
Occasionally	0.98 (0.93, 1.04)	1.15 (1.08, 1.22)^***^	0.90 (0.86, 0.94)^***^	1.11 (1.06, 1.17)^***^
Daily	0.89 (0.81, 0.98)^**^	1.18 (1.08, 1.30)^***^	0.91 (0.83, 1.00)^**^	1.07 (0.98, 1.18)

Values are presented as relative risk (95% confidence interval). NFHS, National Family Health Survey.

1 The results were adjusted for age, marital status, caste, religion, place of residence, schooling, wealth quintile, employment status, and tobacco use.

* p<0.05,

** p<0.01,

*** p<0.001.
